# Variability in Alignment and Bone Resections in Robotically Balanced Total Knee Arthroplasties

**DOI:** 10.3390/bioengineering11080845

**Published:** 2024-08-19

**Authors:** Matthew S. Hepinstall, Catherine Di Gangi, Christian Oakley, Michael Sybert, Patrick A. Meere, Morteza Meftah

**Affiliations:** Department of Orthopedic Surgery, NYU Langone Health, New York, NY 10003, USA

**Keywords:** total knee arthroplasty, robotic-arm assistance, alignment targets, resection targets

## Abstract

Image-based robotic-assisted total knee arthroplasty (RA-TKA) allows three-dimensional surgical planning informed by osseous anatomy, with intraoperative adjustment based on a dynamic assessment of ligament laxity and gap balance. The aim of this study was to identify ranges of implant alignment and bone resections with RA-TKA. We retrospectively reviewed 484 primary RA-TKA cases, stratified by preoperative coronal alignment. Demographics and intraoperative data were collected and compared using Chi-square and ANOVA tests. Planned limb, femoral, and tibial alignment became increasingly varus in a progressive order from valgus to neutral to the highest in varus knees (*p* < 0.001). Planned external transverse rotation relative to the TEA was lowest in the valgus cohort; relative to the PCA, whereas the varus cohort was highest (*p* < 0.001, both). Planned resections of the lateral distal femur and of the medial posterior femur were greater in the varus group compared to neutral and valgus (*p* < 0.001). There were significant differences between cohorts in planned tibia resections, laterally and medially. Varus knees demonstrated higher variability, while valgus and neutral had more metrics with low variability. This study demonstrated trends in intraoperative planned alignment and resection metrics across various preoperative coronal knee alignments. These findings contribute to the understanding of RA-TKA and may inform surgical decision-making.

## 1. Introduction

Robotic arm-assisted total knee arthroplasty (RA-TKA) has gained increasing popularity in recent years [1,2]. Image-based robotic systems construct a three-dimensional model of the knee based on radiographic images, allowing surgeons to preoperatively plan cases [3,4]. Prior to robotics, traditional manual techniques allowed only certain parameters to be set and measured using jigs. The potential of robotic tools to improve the accuracy of component positioning and limb alignment, compared to conventional techniques, is well established in the literature [5,6,7,8,9,10,11]. While the impact of modern RA-TKA on long-term functional outcomes and implant durability remains unknown, emerging evidence suggests that robotic assistance may deliver a faster recovery with decreased pain, decreased narcotic requirements, and improved patient satisfaction compared to traditional manual techniques [12,13,14,15].

It has been proposed that short-term benefits of RA-TKA may relate to more anatomic knee reconstruction with diminished iatrogenic bone and soft tissue trauma as a result of defining and reliably achieving patient-specific alignment targets [16,17,18,19,20]. Robotic arm-assisted TKA enables surgeons to customize component alignment to approximate the patient’s native anatomy, potentially restoring more natural kinematics and minimizing soft tissue releases [21,22]. This personalized approach, informed by emerging alignment philosophies, such as restricted kinematic and restricted inverse kinematic alignment, may particularly benefit from robotic assistance [23,24].

In addition to approximating native anatomy via a preoperative measured resection plan, RA-TKA enables intraoperative adjustments to the planned bone resections based on objective assessment of planned resection gaps. The software allows surgeons to improve gap balancing with small adjustments to the planned resections prior to bone preparation [25,26,27]. These adjustments remain informed by measured resection and alignment targets, enabling patient-specific customization within a surgeon-accepted range. Surgeons obtain quantitative dynamic assessments of alignment and stability prior to bone cuts, prior to component implantation, and prior to closure, thus increasing the probability of a postoperatively stable knee [28].

Preoperative surgical plans, in conjunction with intraoperative landmark tracking, define an operative region used to provide haptic feedback and prevent the saw from crossing defined boundaries. Robotic platforms can allow surgeons to set “guardrails” and alert surgeons when any alignment or resection parameter is either planned or adjusted outside a surgeon’s pre-defined “safe” range [3,4]. Correlations between individual component metrics and patient outcomes remain unclear due to the wide variation in knee morphologies and alignments prior to TKA. Under current state-of-the-art conditions, RA-TKA allows surgeons to intentionally position implants in a multitude of ways on the same knee; conversely, traditional manual jig-based techniques resulted in unintentional variability [25]. Thus, optimal targets for alignment and soft tissue laxity profiles are not well-established.

The objective of this study was to identify normative ranges for surgeon-selected implant alignment and bone resection targets following the intraoperative plan adjustment phase of RA-TKA, stratified by preoperative coronal alignment. Surgeons adopting robotic surgery may consider these data in establishing their own resection targets and boundaries when handling variable anatomy.

## 2. Materials and Methods

### 2.1. Study Population

This retrospective study analyzed data from primary TKA cases performed using cruciate-retaining (CR) implants (Triathlon, Stryker Corporation, Kalamazoo, MI, USA) and a specific robotic-arm assisted platform (Mako, Stryker Corporation, Kalamazoo, MI, USA) between November 2022 and December 2023 at a single urban academic medical center. Intraoperative data for 626 primary TKA cases with the robotic system were collected, with 484 cases meeting the inclusion criteria after excluding those with posterior-stabilized (PS) implants (*n* = 46), constrained polyethylene inserts or augments (*n* = 67), as well as cases with incomplete intraoperative data (*n* = 7) or from surgeons contributing 10 or less cases to the cohort (*n* = 22). The 484 cases were from 481 patients, 3 of which were bilateral (0.6%). The cohort was further divided by preoperative deformity, according to arithmetic hip–knee–ankle (aHKA) angles, into varus (>2 degrees varus), neutral (≤2 degrees varus or valgus), and valgus (>2 degrees valgus) cohorts.

The 9 surgeons contributing cases to this series were board certified, underwent specific training and certification on this robotic platform, and had each worked on more than 30 cases with this robotic system prior to the study period. Alignment targets evolved from seeking neutral mechanical alignment towards a restricted-inverse kinematic functional alignment strategy leading up to the study period. Using a tibia-first workflow, the native anatomy was approximated with a tolerance of up to 4 degrees of varus; this was followed by adjusting the femoral component alignment and resection magnitudes to improve gap balance, while keeping overall limb alignment within 4 degrees of neutral when possible.

### 2.2. Data Collection

**Figure 1:**
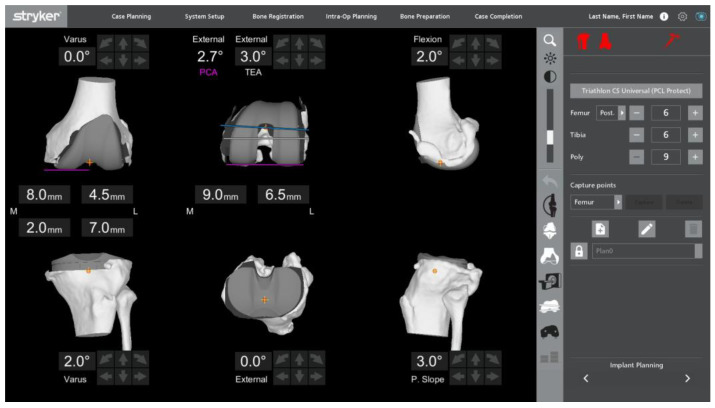
Screen capture from robotic software of the implant planning screen.

Deidentified patient records and procedural data from the robot were collected as part of our institutional quality improvement program, and a human subjects review by our Institutional Review Board (IRB) was obtained prior to assembling and analyzing data for this study. Patient demographic data, including age, sex, race, body mass index (BMI; kg/m^2^), American Society of Anesthesiology (ASA) classification, preoperative diagnosis (osteoarthritis, OA vs. trauma-related vs. rheumatoid arthritis, RA), and smoking status were collected. Preoperative coronal knee alignment was assessed using aHKA measurements based on CT imaging from the robotic software summary screen and used to classify preoperative deformity. The final executed intraoperative resection thicknesses and alignment angles were retrospectively collected from the robotic software. Figure 1 depicts an implant planning screen capture.

### 2.3. Data Analyses

Statistical analyses included Chi-squared, independent samples t-tests, Pearson correlation, and one-way ANOVA tests. When homogeneity of variances was not met, Welch tests were used in lieu of ANOVA. Metric variability was characterized based on the standard deviation (SD) from the mean and categorized into low (≤1.5 mm (mm)/degree) and high (>1.5 mm/degree); the 1.5 threshold was determined by finding the mean of the planned intraoperative variables’ standard deviations.

### 2.4. Patient Characteristics

A total of 191 varus, 189 neutral, and 104 valgus knees were included in the analysis. The baseline demographic data of the study population, stratified into preoperative coronal alignment cohorts, are presented in Table 1. The varus group exhibited a higher proportion of males (37.2%) compared to the neutral and valgus groups (27.5 and 16.3%, respectively, *p* < 0.001). Additionally, a significant difference was found between cohorts for race (*p* = 0.031). Preoperative diagnosis, age at surgery, BMI, smoking status, and ASA classification did not significantly differ between the cohorts.

## 3. Results

Overall, the mean preoperative aHKA was 1.0 degrees varus. The mean planned limb coronal alignment was 1.2 degrees varus, ranging from 4.5 valgus to 6.2 degrees varus. The range of planned coronal alignments were from 4.5 valgus to 4 degrees varus on the femur (mean 0.1 degrees valgus) and 2.0 valgus to 5.1 degrees varus on the tibia (mean 1.3 degrees varus). The mean planned transverse femoral rotation was 1.9 degrees external relative to the TEA and 4.6 degrees external relative to the PCA. Targeted sagittal alignment on the femur had a mean of 4.0 degrees flexion; the mean for tibial posterior slope was 3.3 degrees. The planned mean distal femoral resections, laterally and medially, were 4.7 and 7.2 mm, respectively; for the posterior femur, mean resections were 5.7 and 9.5 mm, respectively. The planned mean proximal tibial resections were 6.1 mm on the lateral and 4.3 mm on the medial side.

### 3.1. Target Alignments and Resection Magnitudes by Demographics

Correlations between demographic variables and planned metrics were assessed. Age at surgery demonstrated a positive correlation with posteromedial femoral resection and the medial sum of resections in flexion (r = 0.123, *p* = 0.007; r = 0.114, *p* = 0.011). Age was negatively correlated with the femoral rotation relative to the TEA (r = −0.131, *p* = 0.004). The magnitude of the associations between age and these metrics was weak. Only femoral coronal alignment was significantly correlated to BMI when assessed (r = 0.091, *p* = 0.045); the strength of this association was also weak.

Sex was statistically significant across alignment cohorts and, thus, was further analyzed with planned metrics. Preoperatively, males in the cohort had a mean aHKA of 2.2 degrees varus, significantly higher than the female mean of 0.5 degrees varus (*p* < 0.001). Mean planned coronal alignment, overall and for the proximal tibia, were significantly more varus in males compared to females (mean 1.7 vs. 1.1 degrees varus, *p* = 0.006; mean 1.6 vs. 1.2 degrees varus, *p* = 0.004). Femoral sagittal alignment was planned in less flexion in males compared to females (mean 3.6 vs. 4.1 degrees flexion, *p* = 0.001). Larger resections were planned for males compared to females for the lateral side of the distal femur and proximal tibia (mean 5.1 vs. 4.6 mm, *p* = 0.003; mean 6.5 vs. 6 degrees varus, *p* < 0.001). Additionally, the sum of resections in extension, both laterally and medially, as well as the medial sum in flexion demonstrated significant differences by sex.

### 3.2. Target Alignments Compared by Preoperative Alignment Category

Table 2 presents intraoperative component alignment and bony resection measurements categorized by preoperative coronal alignment. The mean preoperative aHKA was 4.9, 0.2, and −4.7 degrees for the varus, neutral, and valgus cohorts, respectively (*p* < 0.001). Target coronal limb alignment differed between groups, with increasing varus planned in the varus group compared to neutral and valgus groups (mean 3.0 vs. 0.9 vs. −1.3 degrees varus, *p* < 0.001). Target coronal femoral and tibial alignment also differed between the groups, with increasing varus planned in the varus group compared to the neutral and valgus groups (mean 0.8 vs. −0.3 vs. −1.4 degrees varus; mean 2.2 vs. 1.2 vs. 0.1 degrees varus, *p* < 0.001). Targeted femoral transverse rotation relative to the transepicondylar axis (TEA) and posterior condylar axis (PCA) differed between the groups, with increasing external rotation planned in the varus group compared to neutral and valgus groups (mean 2.4 vs. 2.1 vs. 0.5 degrees; mean 5.0 vs. 4.8 vs. 3.7 degrees, *p* < 0.001). Small observed differences between cohorts for mean planned femoral flexion and the posterior slope of the tibia did not reach statistical significance with the numbers available.

### 3.3. Target Resection Magnitudes Compared by Preoperative Alignment Category

The varus group had larger mean lateral distal femoral and medial posterior femoral resections compared to the neutral and valgus groups (*p* < 0.001). Targets for mean tibia resection, both laterally and medially, were associated with significant differences between cohorts (*p* < 0.001). The varus and neutral cohort demonstrated the same mean magnitude of lateral resection, both greater than valgus (6.3 vs. 6.3 vs. 5.6 mm), whereas valgus had greater medial resection magnitudes compared to neutral and varus (4.9 vs. 4.6 vs. 3.8 mm). The sum of lateral resections in both extension and flexion were larger in the varus group compared to those in neutral and valgus groups (*p* < 0.001, *p* = 0.001). The valgus group had a larger mean medial sum of resections in extension compared to the neutral and valgus groups (*p* < 0.001) whereas the neutral group had the greatest mean medial sum of resections in flexion compared to varus and valgus (*p* = 0.030). There were no statistically significant differences in medial distal femoral or lateral posterior femoral resections among the groups.

### 3.4. Metric Variability

**Figure 2:**
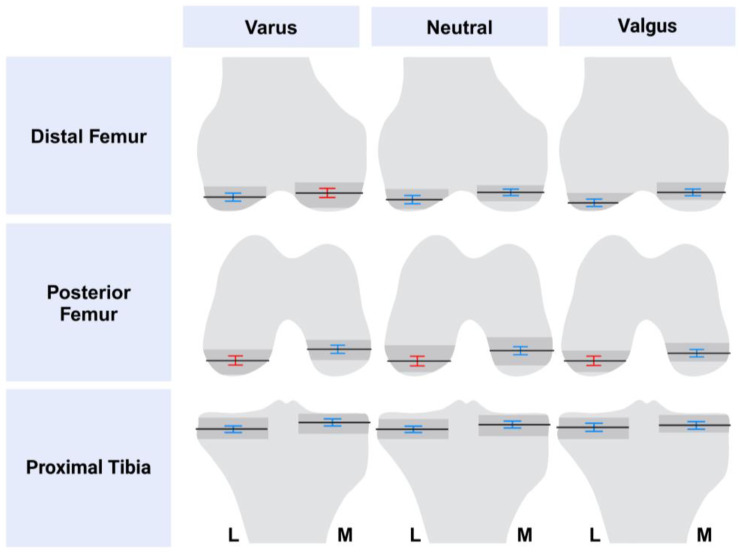
Metric variability in planned resections, stratified by preoperative deformity. Mean resection is shown as a black line. Standard deviations are shown as error bars: blue error bars are low variability; red error bars are high variability. Ranges depicted as darker grey shading. L, lateral; M, medial. Created with BioRender.com.

Metric variability was analyzed according to preoperative alignment. Figure 2 depicts the variability in planned resections.

Varus knees had the highest number of variable metrics, but several metrics were less variable and, thus, were relatively conserved. Low variability was noted in distal femoral alignment, lateral distal femoral resection, medial posterior femoral resection, and femoral flexion. Similarly, low variability was noted in tibia coronal alignment, medial and lateral tibia resections, and tibial slope. Higher variability was seen in medial distal femoral resection, in femoral rotation, and in lateral posterior femoral resections. High variability was also observed in the sum of resections in extension, for both medial and lateral measurements, and the medial and lateral sum of resections in flexion. Overall planned limb alignment demonstrated high variability as well (see Table 2).

Neutral knees had an intermediate number of variable metrics, with more low variability metrics than high. Low variability was seen in overall limb alignment, distal femoral alignment, medial and lateral distal femoral resections, medial posterior femoral resection, and femoral flexion. Low variability was also noted in tibia coronal alignment, medial and lateral tibia resections, and tibial slope. High variability was observed in femoral rotation and lateral posterior femoral resection. Similarly, high variability was seen in the medial and lateral sum of resections in extension, and the medial and lateral sums in flexion.

Valgus knees had the lowest number of high variable metrics of the cohorts. Variability was low for overall limb alignment, distal femoral alignment, medial and lateral distal femoral resections, medial posterior femoral resection, and femoral flexion. Similarly, low variability was noted in tibia coronal alignment, medial and lateral tibia resections, and tibial slope. High variability was observed in femoral rotation and lateral posterior femoral resection. High variability was additionally observed in the medial and lateral sum of resections in flexion and the lateral sum of resections in extension; variability was low for the medial sum of resections in extension.

## 4. Discussion

This study aimed to identify trends in intraoperatively targeted alignment and bony resection metrics during robotically assisted total knee arthroplasty (RA-TKA), stratified by preoperative coronal alignment. Several differences were observed between preoperative varus, neutral, and valgus cohorts in the CR cohort. One notable difference in selected targets between the preoperative alignment cohorts was a neutral to progressively varus planned overall alignment from the valgus to neutral to varus groups, respectively, ranging from −1.3 to 0.9 to 3.0 degrees varus, largely related to variation in proximal tibial alignment. This is consistent with our stated goal to under-correct varus by allowing some residual varus on the tibia, consistent with our described approach to functional planning. Additionally, lateral distal femur and lateral tibial resections were smaller in the valgus group than in the neutral and varus groups, resulting in an increasing lateral sum extension resection in the same fashion. The smaller distal femoral resections in the valgus group suggest that correction of limb alignment was primarily accomplished through a reduction in the distal femoral valgus. Despite no difference in posterior lateral femoral resections, the lateral sum flexion resection showed a significant increase from valgus to varus, largely dependent on differences in lateral proximal tibial resections.

Metric variability was defined and categorized using the standard deviation of the mean to establish a degree of reliability among the planned alignment and resection metrics. Various planned metrics are interdependent on each other and, thus, cannot all be kept constant in variable anatomy. The surgeon’s philosophy and ranking of priorities will ultimately affect which variables have higher or lower variability. Thus, low variability metrics reflect parameters that surgeons choose to keep relatively constant. The lowest variability metrics among the groups were observed in the posterior tibial slope. Low metric variability was additionally observed in all groups regarding distal femoral alignment, femoral flexion, lateral distal femur, medial posterior femur, and medial and lateral tibial resections. The neutral and valgus alignment cohorts exhibited the fewest high-variability metrics, suggesting these knees may be the easiest to balance without adjusting resection angles or magnitudes. The varus group showed the most high-variability metrics and had more variability than other cohorts in planned limb coronal alignment and medial distal femur resection.

There were minor, weak correlations found between age and BMI with planned metrics. We found that males and females demonstrated significant differences in the following planned metrics: coronal alignment, sagittal femur alignment, lateral distal femur resections, lateral tibia resections, sum of resections in extension bilaterally, and the medial sum of resections in flexion. The observed differences in planned coronal alignment likely reflect preoperative alignment differences; we found the mean preoperative aHKA was significantly more varus in males compared to females (2.2 vs. 0.5 degrees varus, respectively). Similarly, a study by Huber et al. of 7456 patients reported a significant difference in mean preoperative aHKA between men and women in their cohort (1.5 vs. 0.7 degrees varus, respectively) [29].

Overall planned limb alignment in our study ranged from 6.2 degrees varus to 4.5 degrees valgus, similar to the range of 6.5 varus to 4.6 valgus described as using functional alignment techniques by Clark et al. [30]. This study does not, however, describe how these ranges change according to preoperative deformity as ours does. A study by Van de Graaf et al. that analyzed differences in planned metrics between alignment philosophies also stratified by preoperative coronal alignment. Functional alignment metrics demonstrated similar trends to the present study: lateral resections for the distal femur and tibia were smallest in the valgus cohort, limb coronal alignment was increasingly varus from valgus to neutral to varus cohorts and was driven by the tibia [31]. Mean medial resections in our dataset were 7.1 to 7.3 mm for the distal femur, 8.6 to 10.0 mm for the posterior femur, and 3.8 to 4.9 mm for the proximal tibia; Van De Graaf et al. reported medial means of 5.7 to 6.0, 8.1 to 8.3, and 5.9 to 6.1 mm for the distal femur, posterior femur, and proximal tibia, respectively [31]. Thus, there was an approximate difference of 1 mm for mean medial resections across deformity cohorts; however, most resection targets were similar in value between this study and ours.

The results of our study align with our understanding of varying knee morphologies in TKA. The smaller lateral compartment resections in the valgus group compared to the neutral and varus groups may be attributed to the lateral femoral condylar hypoplasia seen in valgus knees. The varus cohort exhibited more resections with high variability metrics. To address the lax lateral compartment and tight medial compartment frequently seen with mechanical alignment in preoperative varus knees, tibia-derived overall varus alignment and increased posteromedial femoral resections were observed. Surgeons planned more externally rotated femoral alignments, relative to the TEA and PCA, for varus knees compared to valgus and neutral cohorts.

The neutral alignment groups demonstrated a smaller window of preoperative coronal alignment, implying less asymmetry in medial–lateral ligamentous tensioning and less preoperative bone loss. The relatively low metric variability and intermediate alignment and resection values seen compared to the varus and valgus cohorts are, thus, consistent with expectations.

We have objectively quantified and reported on the choices made by expert surgeons using a robotic platform. These data may serve as a reference of normative values, but they are not meant to be prescriptive as they are not linked to clinical outcomes. Nevertheless, in the absence of evidence-based targets, the data obtained in this study may help guide initial measured resection plans for RA-TKA, potentially minimizing the need for intraoperative adjustments. Pailhe posits that a limitation of robotic systems is the absence of an accepted target, as patient-specific surgical strategies have not yet been defined by the analysis of large datasets [25]. Our data intend to address this by providing a useful guide for surgeons new to RA-TKA, describing the range of alignments and resection magnitudes selected by experienced robotic knee surgeons. Furthermore, these alignment and resection parameters might help surgeons establish guardrails within robotic planning software, alerting surgeons when planned metrics fall outside preestablished ranges.

While these data were derived from RA-TKA, they may also be useful in guiding resection magnitudes during TKA using manual instruments, computer navigation, or custom jigs. As some robotic and computer-assisted surgery platforms do not allow objective assessment of ligament balance before bone resections, these data may help surgeons select alignment targets that minimize the need for soft tissue releases while maintaining limb alignment within 3–4 degrees of a neutral mechanical axis.

For varus knees, the values for distal femur coronal alignment, proximal tibia coronal alignment, femoral flexion, and tibial slope, as well as the resection magnitudes for lateral distal femur, medial posterior femur, medial proximal tibial, and lateral proximal tibial, were relatively conserved. For neutral knees, the values for limb alignment, distal femur coronal alignment, proximal tibial coronal alignment, femoral flexion, and tibial slope, as well as resection magnitudes for the medial and lateral distal femur, medial posterior femur, medial and lateral proximal tibia, were relatively conserved. For valgus knees, the values for limb alignment, distal femur coronal alignment, proximal tibia coronal alignment, femur flexion, and tibial slope, as well as resection magnitudes for the medial and lateral distal femur, medial posterior femur, medial and lateral tibia, and medial extension sum, were relatively conserved. Conserved parameters likely represent appropriate targets during the planning phase, whereas the more variable targets reflect the range of values that we accepted to facilitate soft tissue balancing without compromising acceptable limb alignment.

There were multiple limitations to our study. It was retrospective in nature with a modest number of patients after group stratification. Patients who received PS and constrained implants likely had relatively greater preoperative deformity, introducing selection bias in the larger CR cohort that we examined. Although the accuracy of planned metrics from robotic software to their executed counterparts has been validated with minimal errors in previous studies, our study methodology did not assess for any differences between planned and executed bone resections in our dataset [6,7,11,32]. Clinical outcome data were not available for this dataset and, as such, associations between planned metrics and functional outcomes could not be analyzed. The study is limited to the specific use of one robotic design. A larger planned study will address this deficiency by adding other robots, navigation systems, and manual cohorts to allow for a more comprehensive analysis. Finally, although adequate knee balance was judged during surgery based on joint laxity as being objectively measured by the navigation software, these objective laxity data were not recorded, the force applied was not standardized, and there was no a priori agreement between surgeons regarding acceptable laxity profiles. Furthermore, while these resection parameters were derived using an objective assessment of ligament balance at the time of surgery, future research will be required to determine whether staying within these parameters improves clinical outcomes.

## 5. Conclusions

In summary, our study demonstrated trends in RA-TKA intraoperative planned alignment and resection metrics across various preoperative coronal knee alignments when using a specific cruciate-retaining prosthesis design. These findings may serve as a reference for surgeons new to RA-TKA by allowing them to see how experienced users plan cases. Thus, this study contributes to the understanding of RA-TKA and may inform surgical decision-making during total knee arthroplasty. Future work should investigate associations between targeted metrics and postoperative outcomes to further define optimal ranges.

## Figures and Tables

**Table 1:** Baseline demographics, stratified by preoperative coronal alignment.

	Varus (*n* = 191)		Neutral (*n* = 189)		Valgus (*n* = 104)		
	M ± SD	Range	M ± SD	Range	M ± SD	Range	*p*-Value
Age (years)	67.9 ± 8.9	45–94	67.7 ± 8.9	42–88	67 ± 8.6	45–85	0.716
Male—no. (%)	71 (37.2)	-	52 (27.5)	-	17 (16.3)	-	**<0.001**
BMI (kg/m^2^)	31.4 ± 5.5	18.2–46.2	32.7 ± 5.3	20.8–46.1	31.7 ± 5.8	20.5–47.5	0.064
Preop Dx							0.585
OA	189 (99.0)	-	187 (98.9)	-	103 (99.0)	-	
Trauma	2 (1.0)	-	1 (0.5)	-	-	-	
RA	-	-	1 (0.5)	-	1 (1.0)	-	
Race—no. (%)							**0.031**
White	96 (50.3)	-	99 (52.4)	-	52 (50.0)	-	
Black	29 (15.2)	-	35 (18.5)	-	18 (17.3)	-	
Asian	20 (10.5)	-	19 (10.1)	-	1 (1.0)	-	
Other	46 (24.1)	-	36 (19.0)	-	33 (31.7)	-	
Smoking—no. (%)							0.069
Current	6 (3.1)	-	8 (4.2)	-	2 (1.9)	-	
Former	38 (19.9)	-	58 (30.7)	-	34 (32.7)	-	
Never	144 (75.4)	-	120 (63.5)	-	64 (61.5)	-	
N/A	3 (1.6)	-	3 (1.6)	-	4 (3.8)	-	
ASA Class—no. (%)							0.751
1	6 (3.1)	-	5 (2.6)	-	3 (2.9)	-	
2	109 (57.1)	-	107 (56.6)	-	67 (64.4)	-	
3	75 (39.3)	-	76 (40.2)	-	34 (32.7)	-	
4	1 (0.5)	-	-	-	-	-	
N/A	-	-	1 (0.5)	-	-	-	

M, mean; SD, standard deviation; Dx, diagnosis; OA, osteoarthritis; RA, rheumatoid arthritis; ASA, American Society of Anesthesiologists; BMI, body mass index; kg, kilogram; m, meter, no., number. Ranges are maximum and minimum values.

**Table 2:** Targeted metrics, stratified by preoperative coronal alignment.

	Varus (*n* = 191)		Neutral (*n* = 189)		Valgus (*n* = 104)		
	M ± SD	Range	M ± SD	Range	M ± SD	Range	*p*-Value
Preoperative aHKA (degrees) ^1^	* 4.9 ± 2.2	2.1–12.8	0.2 ± 1.1	−2.0–2.0	* −*4.7 ± 2.1*	−12.5–−2.1	**<0.001**
Planned coronal alignment (degrees) ^1^							
Limb	* 3.0 ± 1.7	−2.0–6.2	0.9 ± 1.4	−4.0–4.4	−1.3 ± 1.4	−4.5–2.2	**<0.001**
Distal femur	0.8 ± 1.2	−3.5–4	−0.3 ± 1.2	−4.5–2.5	−1.4 ± 1.2	−4.5–2.0	**<0.001**
Proximal tibia	2.2 ± 1.1	−1.0–5.1	1.2 ± 1.2	−1.0–4.0	0.1 ± 1.0	−2.0–3.5	**<0.001**
Planned transverse rotation (degrees) ^2^							
Femoral TEA	* 2.4 ± 1.8	−3.0–6.0	* 2.1 ± 1.8	−3.9–6.5	* 0.5 ± 2.0	−5.2–5.0	**<0.001**
Femoral PCA	* 5.0 ± 2.0	−0.8–9.5	* 4.8 ± 2.1	−0.6–9.9	* 3.7 ± 2.0	−0.3–10.0	**<0.001**
Planned sagittal alignment (degrees)							
Femoral flexion ^3^	3.9 ± 1.4	0.0–7.5	4 ± 1.5	0.5–8.0	4.1 ± 1.5	0.0–8.0	0.614
Tibial slope ^4^	3.3 ± 0.6	2.0–5.5	3.2 ± 0.6	2.0–5.8	3.3 ± 0.6	2.0–5.5	0.357
Distal femur resection (mm)							
Lateral	5.5 ± 1.5	0.5–9.5	4.6 ± 1.5	1.0–8.5	3.4 ± 1.4	0.5–7.0	**<0.001**
Medial	* 7.1 ± 1.7	1.5–11.0	7.3 ± 1.2	4.0–10.0	7.3 ± 1.2	4.5–11.0	0.431
Posterior femur resection (mm)							
Lateral	* 5.8 ± 1.7	0.5–10.0	* 5.6 ± 1.8	1.5–11.5	* 5.7 ± 1.7	1.5–9.5	0.429
Medial	10.0 ± 1.5	6.0–13.5	9.5 ± 1.5	4.0–14.5	8.6 ± 1.4	5.5–12.5	**<0.001**
Tibia resection (mm)							
Lateral	6.3 ± 1.2	2.0–10.0	6.3 ± 1.2	2.5–10.0	5.6 ± 1.5	2.0–10.0	**<0.001**
Medial	3.8 ± 1.3	0.5–8.0	4.6 ± 1.3	1.0–9.0	4.9 ± 1.4	1.0–7.5	**<0.001**
Sum of extension resections (mm)							
Lateral	* 11.9 ± 1.9	6.5–17.5	* 10.8 ± 1.8	6.0–15.5	* 9.0 ± 1.8	4.5–14.0	**<0.001**
Medial	* 10.9 ± 2.2	3.5–17.5	* 11.9 ± 1.7	7.0–16	12.2 ± 1.5	8.0–16.5	**<0.001**
Sum of flexion resections (mm)							
Lateral	* 12.1 ± 1.9	5.5–17.5	* 11.9 ± 1.9	6.0–17.0	* 11.3 ± 1.9	6.5–16.0	**0.001**
Medial	* 13.8 ± 1.7	8.0–17.0	* 14.0 ± 1.6	8.5–19.0	* 13.5 ± 1.6	9.5–18.0	**0.030**

M, mean; SD, standard deviation; mm, millimeters; aHKA, arithmetic hip–knee–ankle; TEA, transepicondylar axis; PCA, posterior condylar axis. Ranges are maximum and minimum values. * Standard deviations classified as having high metric variability are italicized. ^1^ Varus measurements are greater than 0, valgus measurements are less than 0. ^2^ External measurements are greater than 0, internal measurements are less than 0. ^3^ Flexion measurements are greater than 0, extension measurements are less than 0. ^4^ Posterior slope measurements are greater than 0, anterior slope measurements are less than 0.

## Data Availability

Restrictions apply to the availability of the data. Data were obtained from NYU Langone Health and requests to view the data can be sent to the corresponding authors.
